# Radiation studies in the Southern Urals—winds of change

**DOI:** 10.1007/s00411-025-01169-5

**Published:** 2025-12-03

**Authors:** Werner Rühm, Andrzej Wojcik, Anna A. Friedl

**Affiliations:** 1 Federal Office for Radiation Protection, Ingolstädter Landstraße 1, 85764 Neuherberg, Germany; 2 MBW Department, Centre for Radiation Protection Research, Stockholm University, Stockholm, Sweden; 3 Department of Radiation Oncology, University Hospital, Ludwig- Maximilians-University LMU Munich, Munich, Germany

## Radiation studies in the Southern Urals—Tailwind

Following the end of Second World War with the atomic bomb explosions over Hiroshima and Nagasaki in 1945 in Japan, two major geopolitical blocks emerged—a block of West European and North American countries led by the United States of America and a block of East European and Asian countries led by the Soviet Union. During the following so-called “Cold War” political tensions developed between the two blocks and persisted until the end of the 1980s, with significant implications for international relations hindering, for example, free economic development, scientific collaboration and personal exchange.

Then, the early 1990s saw a fundamental rearrangement in the international political landscape—a wind of change blew away the political structures that had been established during the Cold War, borders were torn down—both literally between political systems and individual countries, but also mentally between people—and fresh air created an atmosphere of change and forward-looking that stimulated international cooperation and global economic development, in particular scientific and technological collaboration.

For those working in radiation sciences and radiation protection these were particularly exciting times. The above-mentioned changes were already looming on the horizon when the Chornobyl accident happened in 1986 that had a transnational dimension. In the wake of the accident not only areas in Ukraine, Belarus and Russia (all on the territory of the then still existing Soviet Union), but also large European areas outside the Soviet Union were contaminated by various radionuclides released during the accident. It was obvious that an investigation of the consequences of such a large-scale contamination—both in terms of the long-term behaviour of the released radionuclides in the environment and in terms of any radiation-related health consequences to affected populations—required scientific efforts across borders.

Consequently, numerous projects were initiated to study those consequences not only in the vicinity of the Chornobyl reactor but also further away in many countries where nuclear fallout from the accident had been detected. In these projects scientists from whole Europe worked jointly together, exchanged ideas, produced and analysed data, discussed the results, and published their findings. Clearly, it was the wind of change which made these efforts possible and it was the fresh air that allowed scientific collaboration to be put on a new level.

In this new spirit of transparency and openness information on a number of past large-scale radioactive contaminations on the territory of the former Soviet Union became publicly available: For example, in the late 1940s and early 1950s the Techa River in the Southern Urals was contaminated due to releases of liquid radioactive waste from the Mayak Production Association (PA), by then the most important production site for weapons-grade plutonium in the Soviet Union. In 1957, on the site of the Mayak PA a container with radioactive waste exploded and large adjacent areas were contaminated by radioactive fallout (now known as the Kysthym accident). Beginning in the 1950 s, some of the liquid radioactive waste from the Mayak PA was discharged into the close-by lake Karachai—another source of radioactive contamination when the lake dried in 1967 and contaminated sediments were spread by the wind across a broader area. These radioactive releases resulted in radiation exposures of various population groups including people living downstream the Techa River, those living along the East Urals Radioactive Trace where the radionuclides released from the Kysthym accident were finally deposited, those working in the various nuclear, radiochemical, and plutonium facilities on the Mayak PA site, or their families living in the nearby Ozyorsk city.

International scientists who enrolled in post-Chornobyl projects together with institutions from the former Soviet Union immediately recognised the knowledge that their local colleagues had already gained when studying radioecological and dosimetric aspects in those contaminated regions and collecting information on the health status of the affected populations in the Southern Urals. Consequently, as early as 1994 a special issue appeared in “*The Science of the Total Environment*” on “Radiation Exposures in the Southern Urals”, where scientists from the former Soviet Union presented their—albeit still preliminary—findings in a peer-reviewed English-speaking scientific journal. In their preface to this special issue, Werner Burkart and Albrecht Kellerer emphasized that the published articles “*indicate the wealth of the data that have been accumulated since the early 1950s. They also indicate the work that still needs to be done. For these two reasons we consider it extremely important to bring this information into the international science community*” (Burkart and Kellerer 1994). Alexander Akleyev and Eduard Lyubchansky added in their introductory article to the special issue that “*the material presented in this issue cannot cover the full spectrum of problems related to the many different exposure situations in the Urals. However*,* the preliminary results of these studies … are extremely important*,* and the experience gained from the tragedies in the Urals may be of great use for mankind.*” (Akleyev and Lyubchansky 1994).

Consequently, in the 1990s, projects were initiated and funded by various institutions to assess radiation doses of those releases to the affected population, as well as potential radiation-related long-term health consequences. These projects can be seen as a supplement and prolongation to the above-mentioned efforts that had been initiated in the former Soviet Union many years earlier. In two consecutive coordination meetings at Schloß Elmau in Bavaria, Germany, in 1998 and 2000, scientists from the former Soviet Union and the former Western Europe and USA came together to coordinate their efforts and pave the way forward, to “*facilitate the careful planning and the international cooperation that will be required to continue and extend the current efforts*” (Kellerer 2002a). The outcome was summarised in a special issue of this journal, *Radiation and Environmental Biophysics.* The special issue included 18 articles describing studies on the dosimetry and radiation-related health effects among the Mayak nuclear workers, the Ozyorsk population, and the Techa river populations. Additional information was provided on the Semipalatinsk test site including dosimetry and radiation-related health effects in the Altai region and in Kazakhstan. With these findings in mind it became clear that “*a vast new chapter of radiation epidemiology was about to be written*,* and a new base of knowledge was about to be created*” (Kellerer 2002a). These coordination meetings, together with a follow-up workshop on “Radiation Risk Research in the Southern Urals“, which took place in 2002 at lake Chiemsee, Bavaria, Germany, were jointly supported by the US Department of Energy (DoE), the Russian Ministry of Health and Minatom, and the European Commission. These events were instrumental in discussing scientific results already obtained and new ideas for future research projects. All projects, those still ongoing and any planned new projects, were discussed in an open and transparent way of mutual trust among all those involved in the United States, the European Union and the Russia Federation, demonstrating the above-mentioned atmosphere of change and forward-looking that stimulated international scientific collaboration at that time.

In this spirit, various joint projects were initiated and supported already in the 1990s, for example by the European Commission. Two more recent projects were “Southern Urals Radiation Risk Research” (SOUL), funded under the Sixth Framework programme from 2005 to 2009 (https://cordis.europa.eu/project/id/516478), and “Epidemiological Studies of Exposed Southern Urals Populations” (SOLO), funded under the Seventh Framework Programme from 2010 to 2015 (https://cordis.europa.eu/project/id/249675).

Along these lines, beginning in 1994, the US DoE began funding similar but complementary long-lasting projects. Some of those projects continued until 2022, and many of the scientific results obtained have still to be published (see below). All this research has been performed under the Russian Health Studies Program (RHSP) and conducted under the authority of the Joint Coordinating Committee for Radiation Effects Research (JCCRER), a binational committee representing federal agencies in the United States and the Russian Federation. To ensure independent and external scientific oversight, a Scientific Review Group (SRG) was established consisting of renowned scientists from the United States and the Russian Federation in various relevant fields of radiation research, including radiation measurements and dosimetry, radio-epidemiology, and radiation-related health effects. A more detailed overview on the whole programme is given in (Fountos 2017).

## Radiation studies in the Southern Urals—Headwind

Since February 2022, with the beginning of the war in Ukraine, the wind of change has turned again. Almost immediately, the changing international developments significantly affected numerous areas where people had worked jointly together before, and political relationships, economic cooperation, technological development and—not a surprise—also scientific collaboration have continuously worsened.

In the wake of these changing times it was no more possible to continue many of the hitherto fruitful scientific collaborations where institutions from the Russian Federation participated. In this context, one of the many examples concerned the radiation studies in the Southern Urals. In fact, the US DoE terminated their long-lasting support of those studies. What remained to be done was to contribute to an orderly closure of those studies and publish the results that had been obtained so far in the international peer-reviewed literature, to ensure that they become available for an improvement of international recommendations on radiological protection.

From the beginning of the war, the International Commission on Radiological Protection (ICRP) was also directly affected by these changes—in particular because ICRP has members from Ukraine and the Russian Federation who had worked closely together with each other and with colleagues from many other nations, in a joint effort to improve international radiological protection worldwide across national borders. Consequently, ICRP has been closely following the situation and still is, for example, “*concerned about the possibility of harm related to loss of control of nuclear installations and radiation sources*”. Still in 2022 ICRP decided not to “*expend funds in the Russian Federation or Belarus*,* accept funds from Russian or Belarusian institutions*,* or provide financial support for individuals employed by those institutions*”. However, ICRP has encouraged and is still encouraging “*all members and other independent experts to continue to engage in the important work of ICRP for the global public benefit*”. In doing so, ICRP follows their “*Code of Ethics emphasising commitment to public benefit*,* independence*,* impartiality*,* transparency*,* and accountability while maintaining a respectful dialogue*” (ICRP 2022—ICRP Statement on the Conflict in Ukraine, 4 May 2022; https://www.icrp.org/page.asp?id=583).

## Radiation studies in the Southern Urals—the ICRP workshop

Following an initiative of the US DoE, ICRP has recently organized an international workshop on “Thirty Years of Scientific Achievements for International Radiological Protection: Summary of the Southern Urals Health Studies Programme”. The workshop took place on May 24–25, 2024, at the Le Meridien Hotel in Vienna, Austria. More than 500 individuals from more than 60 countries registered for the workshop—about 50 of them were available on site to present and discuss their results, while others presented their results online and participated in the discussions digitally. After the workshop, all presentations were made available through the ICRP website (https://www.icrp.org/page.asp?id=656).

### Contributions of scientists from the United States and the Russian Federation

For 30 years, the United States and the Russian Federation had cooperated in radiation effects research focused on the workers at, and the public around, the Mayak PA in the Southern Urals region north of Chelyabinsk/south of Ekaterinburg. The studies have included about 30,000 monitored workers in the nuclear facilities and over 60,000 members of the affected public in the region, with up to 75 years of multi-generational follow-up.

Given that a continuous funding of RHSP through DoE was no more available after February 2022, the SRG recommended to finish five still ongoing projects including to complete the remaining data analysis and risk model development, and to publish the achieved results. In an effort to summarize the RHSP accomplishments, a one-day workshop should be organized in 2024, a paper summarizing the workshop be published in an open access and peer-reviewed journal, and the remaining RHSP papers should be published in a special issue or theme issue in a relevant international scientific journal.

During the workshop, specific information on those five ongoing projects was provided. Presentations were given jointly by scientists from the United States and the Russian Federation on the following topics: Techa River population dosimetry; Techa River population cancer morbidity and mortality; Mayak worker cancer mortality; Mayak worker dosimetry; and Russian human radiobiology tissue repository.

### Contributions of ICRP members

Since its foundation in 1928, ICRP follows the mission to contribute to an appropriate level of protection against the detrimental effects of ionizing radiation exposure without unduly limiting the benefits associated with the use of radiation. To fulfil this mission, ICRP is continuously reviewing the scientific literature on radiation exposures and radiation-related health effects and is—based on the scientific evidence identified—developing recommendations to enhance radiological protection. While investigations from all over the world are considered in that effort, research performed under the RHSP plays a very important role.

Several task groups under ICRP Committee 1 (“Radiation Effects”) benefit from data from those studies: Task Group 64 (TG64) dealt with radiation risk from incorporation of alpha emitters with significant input from Southern Urals studies for example related to inhalation of plutonium (ICRP 2021). Task Group 91 (TG91) on “Radiation Risk Inference at Low-dose and Low-dose Rate Exposure for Radiological Protection Purposes: Use of Dose and Dose Rate Effectiveness Factors” reviews the scientific evidence relevant for the assessment of radiation risk at low-dose and low-dose-rate including the evidence deduced from Southern Urals epidemiological studies (e.g., Shore et al. 2017). More recently, ICRP launched the Task Group TG119 on “Effects of Ionising Radiation on Diseases of the Circulatory System and their Consideration in the System of Radiological Protection”, and Task Group TG122 on “Update of Detriment Calculation for Cancer”, which both also rely on input from the RHSP studies.

Work under ICRP Committee 2 (“Radiation Dosimetry”) also benefits from those studies. For example, data on dosimetry, bio-dosimetry, and biokinetic modeling from these studies have been and are still being used by Task Groups under Committee 2 to improve for example dosimetry of occupational intake of radionuclides from strontium, americium and plutonium (ICRP 2016, 2019).

### The programme of the workshop

During the workshop, scientists from the United States and the Russian Federation summarized the key scientific advances and findings of the dosimetry and epidemiology studies of their research in the Southern Urals, while scientists involved in ICRP projects described how these results have been incorporated in ICRP recommendations. Finally, areas of potentially productive future research were discussed.

#### Introduction session

- Setting the Scene – Werner Rühm.
- The Role of the Scientific Review Group in the Russian Health Studies Programs: Key Contributions, Influence and Impact on Radiation Protection (US SRG) – Nolan Hertel.
- The Role of the Russian Scientific Review Group in the 30-year Cooperation to Study Radiation Effects in the Southern Ural – Nataliya Shandala.
- JCCRER Research: Aim, Past and Future – Mikhail Sokolnikov.
- Evolution of the Research Performed in the Urals Research Center for Radiation Medicine within the Framework of the Intergovernmental U.S.-Russian Agreement – Alexander Akleyev.
- Short Overview of All RHSP Projects – Bruce Napier.

#### Dosimetry session

- Retrospective Dosimetry of the Emergency Exposed Population of the Ural Region – Basic Concept and Scientific Advances of Project 1.1” – Elena Shishkina.
- Key Results of the Techa River Dose Reconstruction and Possible Future Endeavours – Bruce Napier.
- Reconstruction of Dose to the Residents of Ozersk from the Operation of the Mayak Production Association: 1948–2002 – Bruce Napier.
- Mayak Worker Dosimetry System: History and Perspectives – Alexander Ephimov.
- Supplementary Topics on Mayak Worker Dosimetry – Richard Bull.

#### Epidemiology session

- Stochastic Effects of Environmental Radiation Exposure in Populations Living Near the Mayak Industrial Association – Lyudmila Krestinina.
- Mortality and Cancer Incidence in the Techa River and 1957 Accident Cohorts – Dale Preston.
- 30 Years of International Collaboration on Cancer Risk Estimation in the Mayak Worker Cohort: What Has Been Learned – Dale Preston.
- Effects of Occupational Exposures on Cancer Mortality in the Mayak Worker Cohort – Dale Preston.
- Suggestions for Future Activities – Roy Shore.

#### Panel Discussion/Q&A

- Moderators Benjamin French and Nolan Hertel.

#### Further projects

- Joint Coordinating Committee for Radiation Effects Research (JCCRER): Shorter Term Direction 2 Projects – Scott Miller.
- The Russian Radiobiology Human Tissue Repository in Southern Ural Biophysics Institute FMBA, Russian Federation: Results of Work and Development Prospects – Dariya Oslina.
- Russian Human Radiobiological Tissue Repository: A Unique Resource for Studies of Plutonium-Exposed Workers – Chris Loffredo.
- Future Opportunities: Seversk Pilot Study – Lydia Zablotska.

#### Final Q&A

- Moderators Benjamin French and Nolan Hertel.

#### Scientific achievements and their use in ICRP recommendations

- Overview of ICRP Committee 1 Activities – Importance of Southern Urals Health Studies – Dominique Laurier.
- ICRP *Publication 150*: Cancer Risk from Exposure to Plutonium and Uranium – Richard Wakeford.
- Task Group 91: Radiation Risk Inference at Low-dose and Low-dose Rate Exposure for Radiological Protection Purposes – Werner Rühm.
- Dose Reconstruction and Epidemiological studies in the Southern Urals – Alexander Ulanowski.
- Examples of Studies of Techa River Populations and Mayak Worker Informing ICRP Internal Dose Coefficients – Derek Jokisch.

#### Final remarks

- Data Preservation in the DOE Comprehensive Epidemiologic Data Resource (CEDR) – Ashley Golden.
- Closing – Werner Rühm.

## Radiation studies in the Southern Urals—the new REBS eCollection

As has already been mentioned earlier, this journal—*Radiation and Environmental Biophysics* (REBS)—has supported the publication of some of the first results that were obtained in the course of the joint US-Russian-European research efforts in the Southern Urals: An REBS special issue on “Beyond Chernobyl—a synopsis of the other radio-epidemiological studies in the former Soviet Union” (Kellerer 2002b) documented the outcome of two consecutive coordination meetings held in 1998 and 2000 at Schloss Elmau in Bavaria, Germany (Kellerer 2002c).

Given this earlier involvement of REBS, it is a real pleasure for us as the Editors-in-Chief of this journal that we could offer—with the support of our publisher house Springer Nature—those who presented at the Workshop and their colleagues to publish their results after peer review in the upcoming regular issues of REBS. In parallel, these publications will be bundled in an eCollection on “Radiation Studies in the Southern Urals—Results of a 30 Years Collaboration Between US and Russian Scientists”.

With this initiative we would like to contribute to the dissemination of scientific results produced in an overarching international collaboration that has lasted for several decades and has involved numerous scientists from the United States and the Russian Federation (for many of those scientists the work described at the workshop makes up a significant part of their overall scientific lifetime achievement).

## Radiation studies in the Southern Urals—winds of change

The results presented at this workshop could never have been achieved by just one institution, or just one country. They were only possible because there was an overall international collaboration across borders, political systems, societies, etc.—which unfortunately appears to become more and more difficult these days. It is our responsibility as scientists to reiterate and emphasize again and again how important international cooperation is for scientific progress, and this is particularly true for the work of ICRP.

It is our sincere hope that more of such international scientific collaborations—which strive for an enhancement of international radiological protection and, in more general terms, for an improvement of human wellbeing—will be possible in the future.
